# Mental Health Challenges in Primary Health Care: Assessing Anxiety, Stress, and Psychological Distress Among Medical and Nursing Staff

**DOI:** 10.1192/j.eurpsy.2025.1640

**Published:** 2025-08-26

**Authors:** M. Theodoratou, P. Sklirou, E. Toki, M. Argyrides

**Affiliations:** 1 Social Sciences, Hellenic Open University, Patras, Greece; 2 Psychology, School of Health Sciences, Neapolis University Pafos, Pafos, Cyprus; 3 Speech Therapy, University of Ioannina, Ioannina, Greece

## Abstract

**Introduction:**

Work-related mental health issues are becoming increasingly prevalent, particularly in professions involving direct communication and care, such as healthcare. Primary Health Care (PHC) plays a critical role in prevention, health promotion, and the management of emergency situations. The mental and emotional well-being of healthcare professionals in PHC is essential for maintaining high levels of performance and work effectiveness. However, there is limited research on the psychosocial conditions of healthcare staff in PHC settings, particularly regarding anxiety, stress, depression, and psychological distress.

**Objectives:**

The objective of this study is to evaluate the prevalence of anxiety, stress, depression, and psychological distress among medical and nursing staff working in primary healthcare units in Peloponnese. Additionally, the study aims to examine the associations between these mental health indicators and various individual and professional characteristics, such as age, gender, and professional role.

**Methods:**

An online survey was conducted to gather data from a final sample of 103 healthcare professionals. A questionnaire was constructed ad hoc and comprised two well-established measurement tools. The Depression, Anxiety, and Stress Scale (DASS-21) was employed to assess anxiety, stress, and depression, while the K6+ was used as a self-report measure to evaluate psychological distress.

**Results:**

The results demonstrated that the participants exhibited generally low levels of anxiety, stress, and depression. As indicated by the DASS-21 scale, approximately 75% of respondents reported minimal to no anxiety, with only 7.8% reporting severe anxiety. Similarly, 75% of respondents indicated minimal to no stress, with only 4% reporting severe stress. Regarding depressive symptoms, 76% of participants exhibited minimal to no depressive symptoms, while only 4% demonstrated severe depressive symptoms. However, approximately 20% of the sample exhibited signs of psychological distress, which may indicate a significant mental health concern. Significant correlations were found through statistical analysis: older employees exhibited lower levels of anxiety, while nursing staff demonstrated higher levels of anxiety compared to medical staff. Additionally, women reported higher anxiety levels than their male colleagues.

**Conclusions:**

In conclusion, while anxiety, stress, and depression levels are generally low among healthcare professionals, a notable portion of the workforce is at risk of serious psychological distress. These findings indicate the need for targeted mental health interventions, particularly for younger staff, nurses, and female employees, to ensure the well-being of healthcare professionals and maintain the efficacy of primary healthcare services.

**Disclosure of Interest:**

None Declared

